# Neonatal hyperglycemia induces CXCL10/CXCR3 signaling and microglial activation and impairs long-term synaptogenesis in the hippocampus and alters behavior in rats

**DOI:** 10.1186/s12974-018-1121-9

**Published:** 2018-03-15

**Authors:** Katherine M. Satrom, Kathleen Ennis, Brian M. Sweis, Tatyana M. Matveeva, Jun Chen, Leif Hanson, Akhil Maheshwari, Raghavendra Rao

**Affiliations:** 1 Division of Neonatology, Department of Pediatrics, University of Minnesota, PWB 420 Delaware St SE, Minneapolis, MN 55455 USA; 2 Department of Neuroscience, University of Minnesota, Jackson Hall, 321 Church St SE, Minneapolis, MN USA; 3 Department of Psychology, University of Minnesota, Elliot Hall, 75 E River Rd, Minneapolis, MN USA; 4 Department of Pediatrics, Division of Neonatology, University of South Florida, Tampa General Cir, Suite HMT 450.19, Tampa, Florida 33606 USA

**Keywords:** Prematurity, Blood glucose, Inflammation, Oxidative stress, Neurodevelopment, Hyperglycemia, ELGAN, Hippocampus

## Abstract

**Background:**

Hyperglycemia is common in extremely low gestational age newborns (ELGAN) and is associated with increased mortality and morbidity, including abnormal neurodevelopment. Hippocampus-mediated cognitive deficits are common in this population, but the specific effects of hyperglycemia on the developing hippocampus are not known.

**Methods:**

The objective of this study was to determine the acute and long-term effects of hyperglycemia on the developing hippocampus in neonatal rats using a streptozotocin (STZ)-induced model of hyperglycemia. STZ was injected on postnatal day (P) 2, and littermates in the control group were injected with an equivalent volume of citrate buffer. The acute effects of hyperglycemia on markers of oxidative stress, inflammatory cytokines, microglial activation, and reactive astrocytosis in the hippocampus were determined in the brain tissue collected on P6. The long-term effects on hippocampus-mediated behavior and hippocampal dendrite structure were determined on P90.

**Results:**

On P6, the transcript and protein expression of markers of oxidative stress and inflammatory cytokines, including the CXCL10/CXCR3 pathway, were upregulated in the hyperglycemia group. Histological evaluation revealed microglial activation and astrocytosis. The long-term assessment on P90 demonstrated abnormal performance in Barnes maze neurobehavioral testing and altered dendrite structure in the hippocampus of formerly hyperglycemic rats.

**Conclusions:**

Neonatal hyperglycemia induces CXCL10/CXCR3 signaling, microglial activation, and astrocytosis in the rat hippocampus and alters long-term synaptogenesis and behavior. These results may explain the hippocampus-specific cognitive deficits common in ELGAN who experience neonatal hyperglycemia.

## Background

Hyperglycemia (blood glucose concentration > 150 mg/dL [> 8 mmol/L]) is seen during the first 1 to 2 weeks after birth in 30–80% of extremely low gestational age newborns (ELGAN; birth at < 28 weeks of gestation) [1–3]. A combination of relative hypoinsulinism, peripheral insulin resistance, and glucose infusion for nutrition is responsible for hyperglycemia [4]. Hyperglycemia in this preterm population is associated with increased risk of morbidity and mortality in the neonatal period [5], growth deceleration, and abnormal neurodevelopment that persist until at least 2 years of life [2, 3].

The effects of hyperglycemia on the developing hippocampus are relatively unknown. The hippocampus is central to recognition memory and is vulnerable to injury during a variety of perinatal conditions [6]. Hippocampus-mediated long-term cognitive deficits are common in the ELGAN population. [7] Structural and functional hippocampal deficits have been reported in children with hyperglycemia due to early onset type 1 diabetes (T1D) [8], but whether hyperglycemia has a role in the hippocampal deficits seen in the ELGAN population is not known. Our recent study in rats demonstrates that recurrent hyperglycemia in the neonatal period leads to long-term abnormalities in hippocampal neurochemistry and synaptic architecture that are suggestive of suppressed neuronal activity [9].

In a previous study, using a neonatal rat model, we have demonstrated that recurrent episodes of hypoinsulinemic hyperglycemia upregulate poly(ADP-ribose) polymerase-1 (PARP-1) expression in the cerebral cortex [10], likely due to oxidative stress [11]. There was co-activation of nuclear factor of kappa light polypeptide gene enhancer in B cells (NF-κB) as well as microglial activation. NF-κB is a transcription factor that modulates inflammatory response at the site of injury. [12, 13] The interplay between PARP-1 and NF-κB has been implicated in the upregulation of inflammatory cytokines and microglial activation [14].

Among the chemokines in the brain, C-X-C motif chemokine ligand 10 (CXCL10) and its cognate receptor C-X-C motif chemokine receptor 3 (CXCR3) are important because of the role of CXCL10/CXCR3 signaling in neuronal function and synaptic activity [15]. Both the neurons and glia express CXCL10 and CXCR3 [16, 17]. In hippocampal cell cultures, increased CXCL10 expression alters GABA and glutamate receptor expression and synaptic network activity [18]. Increased CXCL10 levels in the CSF are associated with cognitive deficits in adult humans with CNS infections (e.g., encephalitis) and neurodegenerative conditions (e.g., Alzheimer’s disease) [17, 19–21].

CXCL10/CXCR3 signaling has been implicated in the pathogenesis of T1D, including pancreatic β cell destruction [22–24]. Serum CXCL10 levels are increased in human adults with T1D [25, 26] and in the prediabetes stage in the non-obese diabetic mouse model of T1D [27]. Conversely, hyperglycemia causes CXCL10 release from human monocytes via the Toll-like receptor (TLR) 2 and TLR 4 pathway [26]. Administration of NF-κB inhibitor abrogates CXCL10 release. Whether hyperglycemia affects CXCL10/CXCR3 signaling in the developing hippocampus is not known.

The objective of our study was to determine the acute and long-term effects of hyperglycemia on the developing hippocampus in neonatal rats. The acute effects were determined by measuring the expression of CXCL10/CXCR3, PARP-1, and NF-κB, and their downstream targets, including apoptosis-inducing factor (AIF) and B cell lymphoma 2 (Bcl-2), and NMDA receptor (NR2B) expression. The long-term effects were determined by evaluating hippocampal dendritic architecture using microtubule-associated protein (MAP-2) immunohistochemistry, postsynaptic density protein (PSD) 95 protein expression, and performance in a hippocampus-mediated behavioral test (Barnes maze) at adulthood.

## Methods

### Animals

Experiments were performed with the approval of the Institutional Animal Care and Use Committee of the University of Minnesota. Male and female Wistar rats were studied from postnatal day 1 (P1) to P90 (adulthood). Pregnant dams were purchased (Charles River Laboratories, Wilmington, MA) and housed under standard laboratory conditions on ad lib diet and 12:12 h light and dark cycles.

### Induction of neonatal hyperglycemia

Hyperglycemia was induced using streptozotocin (STZ) (Enzo Life Sciences, Farmingdale, NY), a model that was adapted from previous studies [28–31]. STZ has relative selective cytotoxicity against pancreatic β cells and leads to hypoinsulinemic hyperglycemia. STZ administration in the neonatal period leads to transient hyperglycemia with hypoinsulinism [31], similar to the condition in the human ELGAN. Pilot studies were performed to determine the STZ dose that would cause moderate hyperglycemia comparable to that experienced by the human ELGAN (blood glucose concentration, 270 mg/dL; 15 mmol/L) [1]. Doses between 50 and 100 mg/kg were trialed. The 100-mg/kg dose led to the most consistent hyperglycemia without significant mortality. This dose was used in subsequent experiments. Immediately prior to injection, STZ was dissolved in a citrate buffer (pH 4.5) to achieve a final concentration of 15 mg/mL. Pups were separated from their dams, and 100 mg/kg of STZ was intraperitoneally injected on postnatal day (P) 2 (total volume injected = 50–60 μL). P2 was chosen to represent the ELGAN population, as this time point in rat pups corresponds approximately to the human brain development equivalent to a 24- to 26-week gestation preterm human infant [32, 33]. Littermates in the control group were injected with an equivalent volume of citrate buffer alone. Pups were returned to their dams after injection. Animals were weighed, and glucose measurements were determined in the tail vein blood samples (Accu-Chek®) daily until P6, and on P14 and at adulthood.

To confirm that the effects are due to hyperglycemia and not the administered STZ, a separate set of rats was subjected to recurrent hypoinsulinemic hyperglycemia twice daily from P3 to P12 using a previously described model from our lab [10]. Briefly, the pups were pretreated with octreotide (100 μg/kg, s.c.) to block insulin release from the pancreas, followed by 30% dextrose in a dose of 3 g/kg s.c. Littermates in the control group were injected with octreotide followed by an equivalent volume of 0.9% saline [10]. This model results in hyperglycemia (mean blood glucose ~ 220 mg/dL) of 2-h duration [10].

### Tissue preparation

Rats subjected to STZ-induced hyperglycemia and control rats were killed on P6 or P90 using sodium pentobarbital (100 mg/kg, i.p.). Animals subjected to the recurrent hyperglycemia were killed on P13. The brain was removed, and the hippocampus was dissected on ice, then flash-frozen using liquid nitrogen, and stored at − 80 °C until analysis. Rats used for histochemical analysis underwent in situ transcardial perfusion-fixation with paraformaldehyde before brain removal as in our previous studies [10, 34].

### Quantitative real-time PCR

Transcript expression in the hippocampus was determined using commercial primers (TaqMan, Applied Biosystems, Carlsbad, CA) and a gene expression kit (TaqMan, Applied Biosystems, Carlsbad, CA) as previously described from our lab [34]. Samples (*n* = 6–12/group) were assayed in duplicate using ribosomal protein s18 as control. Some experiments were repeated using GAPDH as control to confirm the observed results.

### Western blot analysis

Protein concentrations of CXCR3 and PSD95 in the hippocampus were determined using 20 μg of hippocampal homogenate and primary antibodies against CXCR3 and PSD95 (1:1000; Abcam, Cambridge, MA) and β-actin (1:5000; Abcam) using previously published methods (*n* = 4–6/group) [34]. Following incubation with corresponding fluorescent secondary antibodies, the membranes were imaged (Odyssey Infrared Imaging System; LI-COR Biosciences, Lincoln, NE). The density of the target protein relative to β-actin was determined.

### Immunohistochemistry

The coronal brain sections (20 μm) corresponding to 0.8 to 2.6 mm anterior to the interaural line in an age-appropriate rat brain atlas [35] were obtained using a cryostat. The choice was based on our recent study demonstrating neurochemical and structural changes in the hippocampus of the corresponding region following recurrent hyperglycemia [9]. On P6, microglial activation in the hippocampus was determined using CD-11 immunohistochemistry (1:300; Abcam) as previously described [10]. A chromagen kit (Vector Laboratories) was used to visualize the protein/antibody complex, and all CD11 cells in the hippocampus were counted at × 10 magnification using ImageJ software (ImageJ, US National Institutes of Health, Bethesda, MD, USA; https://imagej.nih.gov/ij/) and the total number of cells/mm^2^ was determined (*n* = 6 per group, two slides per animal). Anti-S100β protein (1:200; Abcam) was used to quantify immunoreactive astrocytes in the hippocampus, which were counted in a similar manner at × 20 magnification (*n* = 6 per group, two slides per animal) as the microglia.

The cellular origin of CXCL10 (Bioss 1:100) was determined by colocalizing with GFAP, NeuN, and Iba1. The cellular origin of CXCR3 (Novus 1:100) was determined by colocalizing with NeuN (neuron, Millipore 1:100), Iba1 (microglia, Wako 1:1000), and GFAP (astrocyte, Novus 1:200) using previously described methods [34].

On P90, the dendritic structure of the hippocampus was determined by staining for microtubule-associated protein-2 (MAP-2) as previously described [36]. The mean integrated density of MAP-2 fluorescence in the CA1 region was determined using a software program (Adobe Photoshop, San Jose, CA) as described in our previous study [37].

### Neurobehavioral testing

#### Barnes maze

**Fig. 1:**
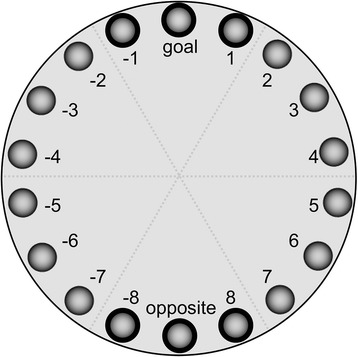
Barnes maze schematic demonstrating 18 evenly spaced holes around the perimeter of the platform with a goal location indicated at the top and an opposite hole at the bottom of the maze

STZ (*n* = 18) and control (*n* = 21) rats were tested for hippocampal function on P90 for a total of 7 days during the light phase (6 am to 6 pm) on the Barnes maze [38]. The elevated platform was circular, black, 0.95 m in diameter, with 18 evenly spaced 10-cm holes placed around the edge. A black escape chamber served as the goal location and was placed under one of the holes (see Fig. 1). This goal location remained in the same position in relation to the spatial cues of the room throughout the entire experiment. All training and probe trials were video recorded, and the animal was tracked using a software program (AnyMaze, Stoelting Co. Wooddale, IL).

The rats were habituated to the maze prior to testing, during which time they were allowed to explore the maze and were guided to the goal location to enter the escape box. Rats were trained on the Barnes maze for 3 days followed by 1 day of probe testing (probe 1). Then they were trained for an additional 2 days followed by probe 2. The rats in both groups were trained for three trials per day with an inter-trial period of 5 min. The maze and goal location chamber were cleaned with 70% ethanol between each trial to eliminate odor cues. Each trial began with the lights off in the room and the animal placed in the center of the maze beneath an opaque container.

To begin the trial, bright overhead lights were turned on to serve as an aversive stimulus, the opaque container was removed, and the rat was given 3 min to explore the maze and find the goal location [39]. If the rat did not reach the goal location during the allotted 3 min, it was guided to enter the goal location after the trial period has ended. Once it entered the dark chamber, the lights were turned off and the animal was allowed to stay in the goal location while the maze was being cleaned for the next trial. Probe testing was a single 90-s trial similar to the previous training days, except the goal location was covered (instead of the hole leading to the dark chamber). Head pokes into each of the 18 holes as the animal explored the maze were measured. During probe testing, head pokes into the goal hole and the holes adjacent to the goal hole (goal hole, hole 1, and hole − 1; Fig. 1) were grouped together and categorized as “good guesses” while those near the opposite side (opposite hole, hole 8, and hole − 8; Fig. 1) were categorized as “bad guesses.” For each animal and each probe session, error rates were calculated as (good guess pokes)/(total head pokes) or (bad guess pokes)/(total head pokes) in order to capture polar spatial memory retention.

### Statistical analysis

All data are reported as mean ± SEM. The control and hyperglycemia groups were compared using two-tailed unpaired *t* tests (GraphPad Prism, La Jolla, CA). Barnes maze data were analyzed using mixed-model multifactorial ANOVA with day and treatment as independent factors and rats as a random event variable. Statistical significance was set at alpha < 0.05.

## Results

### STZ induces moderate neonatal hyperglycemia and growth restriction in rats

Hyperglycemia was observed by 24 h after the STZ injection with a mean blood glucose concentration of 265.4 ± 14.5 mg/dL on P3. Hyperglycemia was sustained until P6, at which time the mean glucose value was 267.1 ± 27.6 mg/dL. The mean blood glucose concentration from P3 to P6 was 144.1 ± 2.5 mg/dL in the control group. All these intergroup differences were statistically significant, *p* < 0.01. By P14, the difference between STZ and control animals’ blood glucose values were less pronounced but still significant (185.0 ± 4.5 mg/dL vs. 163.0 ± 2.3 mg/dL, *p* = 0.002).

By chance, the pups randomized to the STZ group were 12% lighter on P2 compared with those randomized to the control group (*p* < 0.05). Subsequently, the animals in the STZ group continued to remain 20–24% lighter until P6 (Table 1).

**Table 1:** Mean daily weights comparing control and STZ groups

Postnatal day	Control group mean body weight (g)	STZ group mean body weight (g)	Difference between groups (%)
2	8.3 ± 0.14	7.3 ± 0.14	12.0
3	10.3 ± 0.27	7.8 ± 0.19	24.3
4	12.2 ± 0.24	9.8 ± 0.24	19.7
5	13.8 ± 0.26	11.0 ± 0.25	20.3
6	15.9 ± 0.34	12.2 ± 0.23	23.3

Values are mean ± SEM, *n* = 21–65/group

On P90, there was no difference between the control and STZ groups in blood glucose levels after overnight fasting (123.0 ± 7.5 mg/dL vs. 142.4 ± 9.3 mg/dL, *p* = 0.1). However, the STZ group had higher blood glucose levels compared with the control group in the fed state (286.1 ± 68.5 mg/dL vs. 152.5 ± 3.1 mg/dL, *p* < 0.05). Male rats in the STZ group had a lower body weight on P90 compared with the control group (404.5 ± 13.9 g vs. 485.8 ± 5.7 g, *p* = 0.0002). This difference was not present for female rats (248.2 ± 23.4 g vs. 271.8 ± 4.4 g, *p* = 0.37).

The mean blood glucose concentration in the recurrent hypoinsulinemic hyperglycemia model was 217 ± 17 mg/dL (vs. control, 128 ± 2 mg/dL, *p* < 0.05).

### Neonatal hyperglycemia upregulates markers of oxidative stress and inflammation in the hippocampus on P6

The transcript expression of PARP-1 was upregulated by 58% and that of NF-κB was upregulated by 276% in the STZ group relative to the control group (*p* < 0.05). Transcript expression of AIF was not altered, while Bcl2 was upregulated 60% and NR2B mRNA expression was downregulated 40% in the STZ group (Table 2).

**Table 2:** Relative mRNA expression of upstream and downstream effectors of CXCL10/CXCR3 signaling on P6

Transcript name	Gene ID	Description	Control group	STZ group
PARP-1 (*n* = 6)	Parp1	Poly(ADP-ribose) polymerase 1	1.0 ± 0.17	1.6 ± 0.13*
NF-κB (*n* = 6)	Nfkb1	Nuclear factor of κ light polypeptide gene enhancer in B cells 1	1.0 ± 0.10	2.8 ± 0.18*
BCL2 (*n* = 12)	Bcl2	B cell leukemia/lymphoma 2	1.0 ± 0.06	1.6 ± 0.07*
AIF (*n* = 7)	Aifm1	Apoptosis-inducing factor	1.0 ± 0.10	1.1 ± 0.12
NR2B (*n* = 6)	Grin2b	Glutamate receptor, ionotropic, NMDA2B	1.0 ± 0.10	0.6 ± 0.05*

Values are mean ± SEM, *n* = 6–12

*p < 0.05

**Fig. 2:**
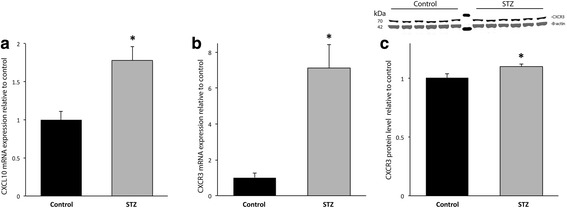
The acute effect of STZ-induced hyperglycemia on the expression of CXCL10 and its receptor CXCR3 on P6. Black, control group; gray, hyperglycemia group, *p < 0.05. **a** CXCL10 mRNA expression was increased by 78%. **b** CXCR3 mRNA expression was increased by sevenfold in the hyperglycemia group, relative to the control group. **c** CXCR3 protein expression was increased by 10%

**Fig. 3:**
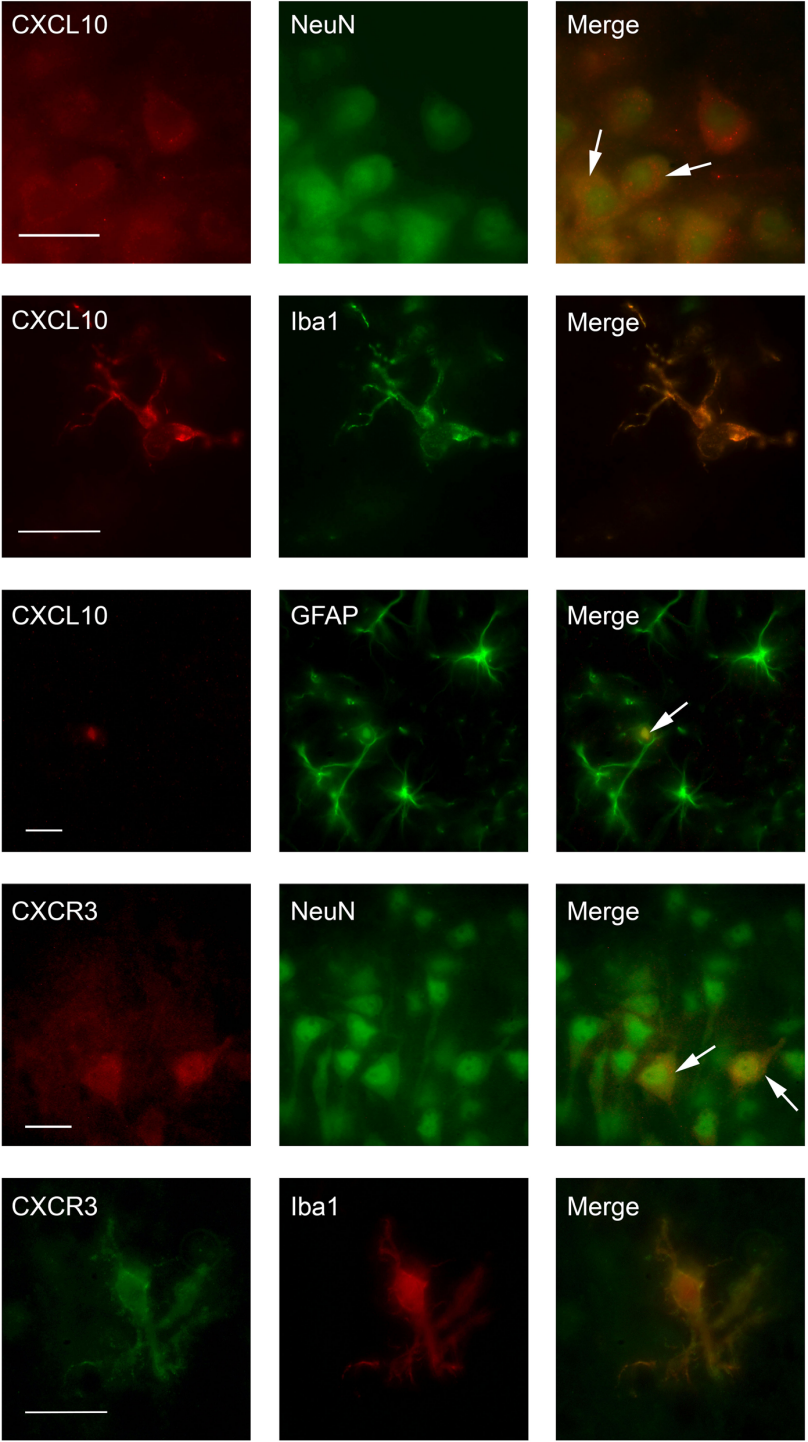
CXCL10 colocalizes with neurons, microglia, and astrocytes; CXCR3 colocalizes with neurons and microglia. Brain sections from the hippocampal CA1 region positive for CXCL10 and NeuN (with merge image), CXCL10 and Iba1 (with merge image), CXCL10 and GFAP (with merge image), CXCR3 and NeuN (with merge image); and CXCR3 and Iba1 (with merge image). Scale bars = 25 μm

Compared with the control group, the expression of CXCL10 and its receptor CXCR3 mRNA transcripts were upregulated in the STZ group (Fig. 2a, b). Repeating the CXCR3 qPCR experiments using GAPDH as a reference gene demonstrated a similar degree of upregulation (STZ group, 6.0 ± 1.5 a.u.; control group, 1.0 ± 0.32 a.u., *p* < 0.05). CXCR3 protein expression was 10% higher in the STZ group (Fig. 2c). CXCL10 colocalized with neurons, astrocytes, and microglia (Fig. 3). CXCR3 strongly colocalized with neurons and microglia, but not with astrocytes (Fig. 3).

CXCL10 and CXCR3 transcript expression were upregulated by 97% (1.97 ± 0.36 a.u. vs. 1.0 ± 0.11 a.u., *p* < 0.05) and 442% (4.42 ± 1.06 a.u. vs. 1.0 ± 0.08 a.u., *p* < 0.05), respectively, in the recurrent hyperglycemia group compared with the control group.

### Neonatal hyperglycemia causes microglial activation and astrocytosis in the hippocampus on P6

**Fig. 4:**
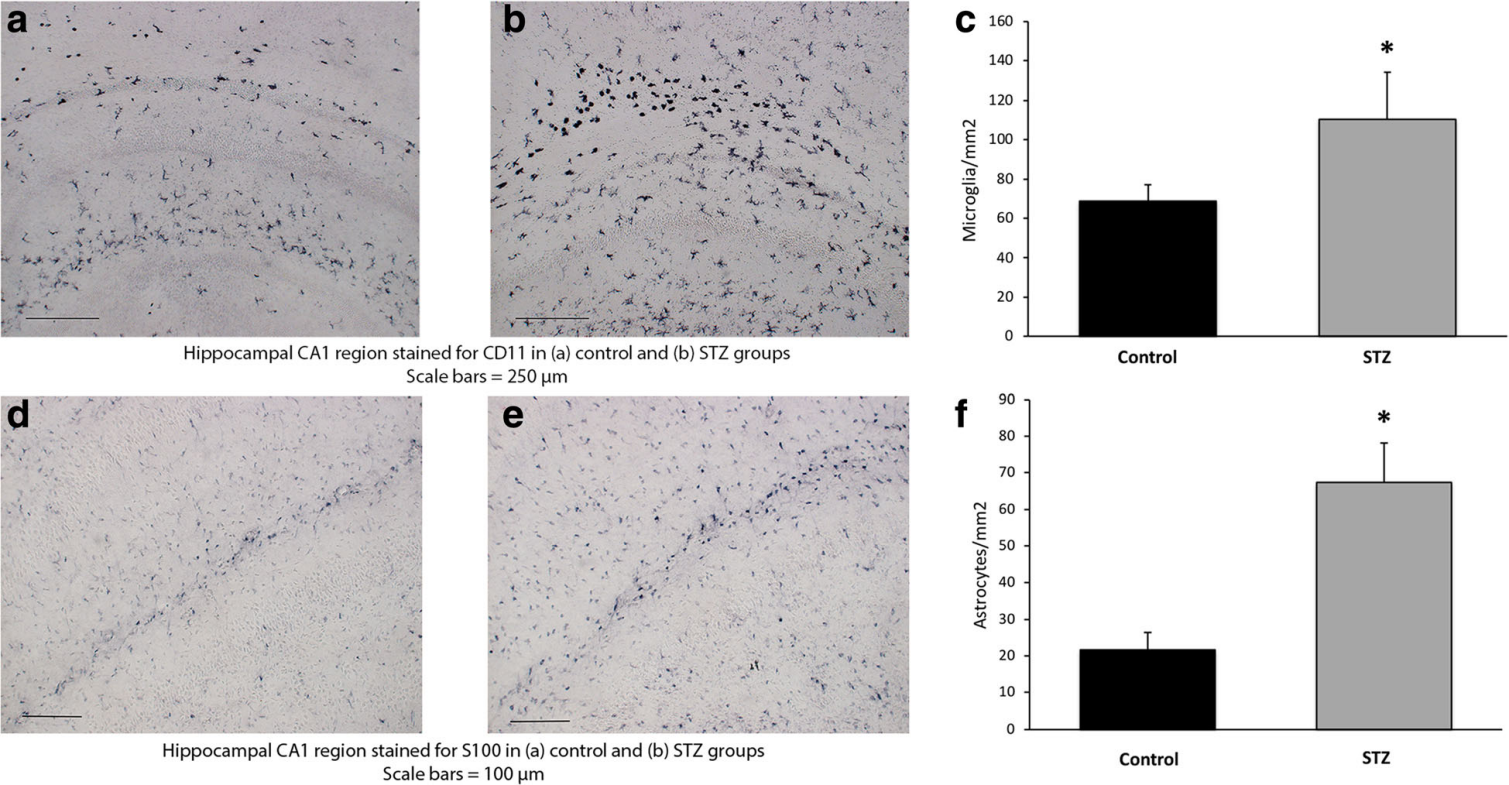
The acute effect of STZ-induced hyperglycemia on microgliosis and astrocytosis on P6. Representative photomicrographs of the hippocampal CA1 region at 10× stained for CD-11 from **a** control and **b** hyperglycemia groups. Scale bars = 250 μm. The number of CD-11-positive microglia was significantly increased in the hyperglycemia group (gray) relative to control (black), *p < 0.05 (**c**). Representative photomicrographs of the hippocampal CA1 region at 20× stained for S100β astrocytes from **d** control and **e** hyperglycemia groups. Scale bars = 100 μm. The number of S100β positive astrocytes was significantly increased in the hyperglycemia group (gray) relative to the control (black), *p* < 0.05 (**f**)

Relative to the control group, more CD11-positive cells, representing microglia, were present in the hippocampal CA1 region in the STZ group (control group, 68.62 ± 11.37 cells/mm2; STZ group, 110.28 ± 15.12 cells/mm^2^, *p* < 0.05; Fig. 4). In a similar manner, the number of S100β immunoreactive astrocytes was increased over threefold in the STZ group, relative to the control group (control group, 21.55 ± 4.88 cells/mm^2^; STZ group, 67.44 ± 10.77 cells/mm^2^, *p* < 0.05; Fig. 4).

### Neonatal hyperglycemia leads to long-term hippocampus-based deficits in learning and memory

**Fig. 5:**
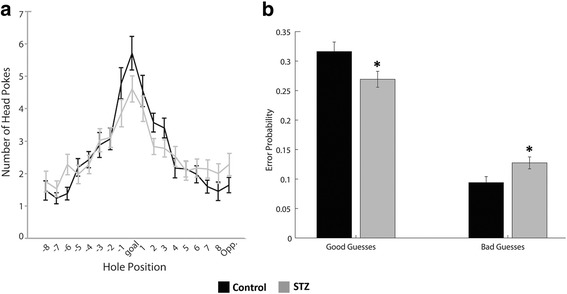
The effect of STZ-induced hyperglycemia on long-term hippocampal function as measured by the Barnes maze behavioral test on P90. Hole pokes made by animal on the maze during probe trials (schematic is shown in Fig. 1), with goal location represented in the center and subsequent holes moving progressively away from the goal (**a**). Quantified “good guesses” represented by hole pokes nearest the goal location compared with “bad guesses” represented by hole pokes furthest from the goal location, demonstrating that hyperglycemia group (gray) made fewer “good” and more “bad” guesses compared with the control group (black) (**b**), *p < 0.05

Analysis of performance in the Barnes maze demonstrated a significant overall main effect of guess type (good guess vs. bad guess) (*F*(1, 39) = 163.92, *p* < 0.0001) after collapsing across both probe sessions. While there was no significant main effect of hyperglycemia on guess rate (*F*(1, 39) = 0.49, *p* = 0.48), there was a significant interaction between hyperglycemia and guess type (*F*(1, 1) = 9.31, *p* < 0.01). Rats in the STZ group made fewer “good guesses” and more “bad guesses” (Fig. 5).

### Neonatal hyperglycemia leads to decreased dendritic density in the adult hippocampus

**Fig. 6:**
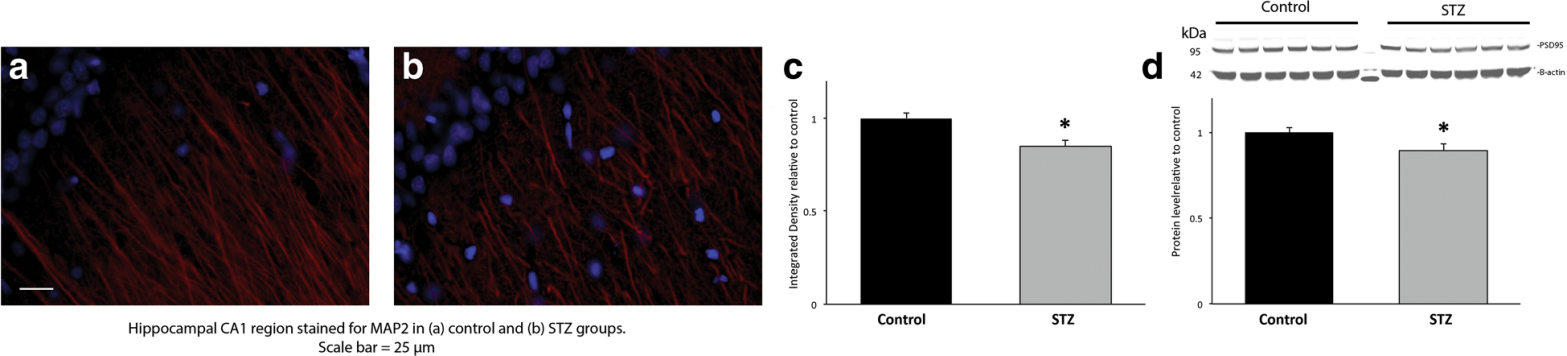
The long-term effect of STZ-induced hyperglycemia on synaptogenesis on P90. Representative fluorescent photomicrographs of the hippocampal CA1 region immunostained for MAP2 (red) in **a** control and **b** hyperglycemia groups. Scale bars = 25 μm. Representative micrographs demonstrate qualitatively disorganized and truncated dendrites in the formerly hyperglycemic hippocampus compared with the hippocampus of the control rat. **c** The quantified average integrated density of fluorescent staining was decreased by 16% in the hyperglycemia group (gray) relative to the control group (black), *p < 0.05. **d** Protein expression of PSD95 in the hippocampus on P90 was decreased by 11% in the hyperglycemia group (gray) relative to the control group (black), *p < 0.05

The effect of neonatal hyperglycemia on the dendritic structure in the CA1 region of the adult hippocampus is shown in Fig. 6. Representative micrographs demonstrate qualitatively disorganized and truncated dendrites in a formerly hyperglycemic rat hippocampus compared with a control rat (Fig. 6a, b). Quantitatively, there was a 16% decrease in integrated MAP-2 density in the STZ group compared with the control group (*p* < 0.05; Fig. 6c). The protein expression of PSD95 was decreased by 11% in the STZ group vs. the control group (*p* < 0.05; Fig. 6d).

## Discussion

Understanding how high glucose levels affect the developing brain is important because it impacts clinical decision-making. Currently, treatment of hyperglycemia in preterm infants is highly variable and controversial and involves restricting glucose infusion and/or insulin therapy [40]. Better evidence of how hyperglycemia impacts neurodevelopmental outcomes will help neonatal providers make better-informed treatment decisions.

Our study demonstrates that hyperglycemia comparable in severity to that experienced by the ELGAN is associated with growth deceleration and evidence of oxidative stress and inflammation in the hippocampus in the neonatal period, as well as long-term negative effects on hippocampal dendrite development and function in adulthood in rats. These findings are consistent with clinical data reporting restriction of somatic and brain growth [2] and impaired neurodevelopment in preterm infants with a history of hyperglycemia in the neonatal period [3]. The data are also consistent with previous studies showing an association between recurrent hyperglycemia and oxidative stress and microgliosis in the cerebral cortex and hippocampus of neonatal rats [10, 11].

We sought to use an animal model of hyperglycemia that was similar in etiology, severity, and duration to that seen in the ELGAN population. Although hyperglycemia experienced by these extremely preterm infants is multifactorial in nature [1], our STZ model addresses a major component of this phenomenon, namely functional hypoinsulinism. The neonatal STZ model takes advantage of the drug’s relative selectivity for pancreatic β cells via uptake by the glucose transporter 2 (GLUT2) at the cell membrane. Extra-pancreatic effects of systemically administered STZ are limited to the liver and kidney, which also express GLUT2. [41] There are no direct CNS effects since the blood-brain barrier does not express GLUT2 [42]. Therefore, we are confident that the hippocampal structural and functional effects are due to hyperglycemia and not STZ itself. Similarities between the results of the STZ model in the present study and those in our previous [10] and current non-STZ recurrent hyperglycemia model corroborate this contention. By inducing hyperglycemia between P2 and P6, we were able to probe the effects on the preterm brain, as the rat pup brain on P2 is developmentally equivalent to a human preterm brain at approximately 24 to 26 weeks gestation and the P6 pup brain being a developmental approximate to a 32-week gestation human brain [32, 33].

Our previous study demonstrated that hyperglycemia is associated with PARP-1 and NF-κB upregulation and microgliosis in the cerebral cortex [10], likely in response to oxidative stress [11]. AIF expression was not altered, while Bcl2 expression was upregulated, suggesting lack of cell death. Our present study confirms a similar response in the hippocampus and demonstrates the novel finding that hyperglycemia upregulates the CXCL10/CXCR3 signaling pathway. Although this pathway is implicated in the pancreatic β cell destruction in T1D and serum CXCL10 is considered a biomarker of T1D in adult humans [43], to our knowledge, this is the first demonstration of CXCL10/CXCR3 upregulation in the hippocampus due to hyperglycemia. Upregulation of these markers in the recurrent hyperglycemia model rules out STZ as the primary cause of CXCL10/CXCR3 upregulation. CXCL10/CXCR3 upregulation was likely mediated through NF-kB. Consistent with this possibility, a previous study demonstrated that blockage of NF-kB with IkB abrogates hyperglycemia-induced CXCL10 release from monocytes [26]. However, a feed-forward effect of CXCL10/CXCR3 on PARP1 and NF-kB expression cannot be ruled out [44]. Our evidence that CXCR3 is expressed in the neurons and microglia, and CXCL10 in all three cell types (neurons, astrocytes, and microglia) during the acute phase is consistent with previous studies [16–18, 45], and suggests that all three cell types are the likely source of CXCL10 and that neurons and microglia are the likely targets of CXCL10 action. The increased number of microglia in the STZ group in the absence of an upregulation of apoptotic factors suggests that microglia may play a neuroprotective role in facilitating tissue repair and preventing further injury [46]. The increased number of astrocytes in the STZ group may represent a developmental difference in astrocyte response to dysglycemia, as some of the adult diabetes models have observed a decrease in activated astrocytes in the setting of hyperglycemia [47].

Abnormal CXCL10/CXCR3 signaling may be responsible for the decreased synaptic density and abnormal performance in Barnes maze at adulthood, likely by altering NMDA receptor expression [18] and glutamatergic neurotransmission. NR2B, a subunit of NMDA receptors, plays an important role in synaptogenesis and learning and memory performance in the hippocampus [48]. NR2B antagonists alter long-term potentiation (LTP) and long-term depression (LTD) in the hippocampi of mice [49]. NR2B transcript expression was 40% lower in the STZ group on P6. In support of abnormal glutamatergic neurotransmission, we have recently demonstrated that recurrent hyperglycemia in neonatal rats is associated with long-term impairments in glutamate-glutamine cycling between neuron and glia in the hippocampus [9].

Animals in the STZ group had higher blood glucose concentration in the fed state at adulthood. This is consistent with previous data in this model [50]. While hyperglycemia in adulthood may explain some of the structural and functional effects [51], it does not negate the significance of our results in the context of ELGAN population who are also at increased risk for metabolic disease including impaired insulin sensitivity as adults [52]. The evidence for insulin resistance appears as early as 2 years of age [53].

Although our model was well suited for assessing the effects of hyperglycemia, it did not account for the other components of neonatal hyperglycemia in human preterm infants, including dextrose infusion and treatment with insulin. In adults with diabetes, glucose fluctuations have a greater triggering effect on oxidative stress than chronic sustained hyperglycemia [54], although the data have been equivocal in preterm infants [55]. Another limitation of the study was the difficulty in determining the sequence of changes among the various transcript and protein expressions. However, in a chronic hypoxia injury model, data support CXCL10 expression is downstream of NF-κB signaling [56]. An exact pathway analysis without appropriate knock-out models, blocking agents, or cell culture experiments cannot be determined. Probing additional markers of oxidative stress would have also strengthened this study and supported this pathway analysis, although conventional markers of oxidative stress have previously been reported in the STZ and recurrent hyperglycemia models. Rosa et al. used a neonatal STZ model of hyperglycemia and demonstrated increased production of superoxide anion and NADPH oxidase activity and an increase in lipid peroxidation in the neonatal rat brain following STZ-induced hyperglycemia [57]. Similarly, Tayman et al. demonstrated increased total oxidant status, xanthine oxidase, and malondialdehyde, and decreased total antioxidant status in the brain tissue of rats after recurrent neonatal hyperglycemia [11].

Finally, this study focused on the hippocampus and hippocampus-mediated learning. However, other functional deficits are also common in extremely preterm infants, for example, deficits in executive function [58, 59]. As mentioned previously, we have demonstrated that recurrent hyperglycemia leads to oxidative stress (PARP-1, NF-κB upregulation) and microgliosis in the cerebral cortex, the brain region important for executive function [10]. Future studies are necessary to explore the full extent of hyperglycemia’s effect on the brain regions.

## Conclusion

In summary, we found that hyperglycemia in newborn rats induces CXCL10/CXCR3 signaling, microglial activation, and astrocytosis, and alters long-term synaptogenesis and function in the hippocampus. Therefore, efforts at minimizing neonatal hyperglycemia should be considered in the ELGAN, so as to reduce the risk of adverse neurodevelopment, in a patient population that is already vulnerable to brain injury from additional comorbidities.

## Abbreviations

AIF: Apoptosis-inducing factor
Bcl-2: B cell lymphoma 2
CXCL10: C-X-C motif chemokine ligand 10
CXCR3: C-X-C motif chemokine receptor 3
ELGAN: Extremely low gestational age newborns
GLUT2: Glucose transporter 2
IBA1: Ionized calcium-binding adapter molecule
LTD: Long-term depression
LTP: Long-term potentiation
MAP-2: Microtubule-associated protein
NF-κB: Nuclear factor of kappa light polypeptide gene enhancer in B cells
NR2B: NMDA receptor
P(x): Postnatal day x
PARP-1: Poly(ADP-ribose) polymerase-1
PSD: Postsynaptic density protein
STZ: Streptozotocin
T1D: Type 1 diabetes
TLR: Toll-like receptor
